# Adsorption of Ethylenediaminetetraacetic Acid on a Gel-Type Ion-Exchange Resin for Purification of Liquid Waste Containing Cs Ions

**DOI:** 10.3390/polym11020297

**Published:** 2019-02-11

**Authors:** Jongho Kim, Chan Woo Park, Kune-Woo Lee, Taek Seung Lee

**Affiliations:** 1 Organic and Optoelectronic Materials Laboratory, Department of Organic Materials Engineering, Chungnam National University, Daejeon 34134, Korea; jh-kim@cnu.ac.kr (J.K.); nkwlee@cnu.ac.kr (K.-W.L.); 2 Decontamination and Decommissioning Research Division, Korea Atomic Energy Research Institute, Daejeon 34057, Korea; chanwoo@kaeri.re.kr (C.W.P.); nkwlee@kaeri.re.kr (K.-W.L.)

**Keywords:** ethylenediaminetetraacetic acid, ion exchange resin, Cs ions, radioactive liquid waste, adsorption

## Abstract

Because of its excellent chelating property, ethylenediaminetetraacetic acid (EDTA) is used as a complex agent, not only for heavy metals, but also for radioactive isotopes during the decontamination of nuclear facilities. The removal of EDTA was investigated by adsorption with commercially available, gel-type, anion-exchange resins (AERs), which are based on cross-linked polystyrene with positive tertiary amine groups. Because of the positive charge on AERs, they could adsorb EDTA effectively even in a solution mixed with ions of cesium (Cs) via electrostatic attraction. Because EDTA adsorption by cation-exchange resins (CERs) was not possible, it was concluded that the negative charges on CERs do not contribute to the interaction with EDTA. The maximum adsorption capacity (*q*_max_) of AER (2 g/L) for EDTA removal, calculated by the Langmuir isotherm model was 0.47 mmol/g for initial EDTA concentrations in the range of 0.01–1 mM in the EDTA/Cs mixed solution. The Langmuir isotherm model was found to be suitable for EDTA adsorption on AERs, indicative of monolayer adsorption. The results clearly suggested that the AERs could efficiently remove EDTA, regardless of the presence of nuclides, such as Cs ions in the aqueous solution.

## 1. Introduction

Because of its excellent chelating ability, ethylenediaminetetraacetic acid (EDTA) has been used as an adsorbent of heavy metal ions generated from the industrial processes [1,2], and in environmental pollutants (e.g., NO_x_ and SO_2_) that cause acid rain and smog [3,4,5,6,7]. In addition, EDTA has been used for rinsing radioactive isotopes in nuclear facility decontamination [8]. Most of the radioactive liquid wastes (RLWs), that are generated during the operation and from the decommissioning of nuclear power plants, usually contain a high EDTA concentration, with various kinds of surfactants [9,10]. The EDTA contained in the RLWs exists as a complex with radioactive isotopes, which makes EDTA difficult to remove by conventional water treatment techniques, such as chemical precipitation and filtration. In particular, it has been reported that more than 10 ppm EDTA could not be separated by the conventional chemical process [11]. Thus, a method of removing EDTA from the RLWs is urgently required for their purification and volume reduction, which are issues that affect the disposal cost of radioactive wastes.

Although various EDTA removal techniques have been reported, including biodegradation [12] and degradation using chlorine [13], they have a shortcoming related to a slow removal rate. The adsorption technique has been applied to separate EDTA from liquid waste using various adsorbents, such as metal oxide [14,15,16], goethite [17], mesoporous silica [18], and activated carbon [19]. The treatment of liquid wastes, containing EDTA by adsorption using activated carbon, is commonly used in the industrial processes, but presents several disadvantages, such as high hygroscopicity, a decrease in pore size during use, and combustion at high temperature after use. The use of zeolite-based adsorbents for the removal of organic contaminants from liquid waste has been reported [20,21]; however, zeolites have confined micro-pores to separate large molecules dissolved in liquid waste. In addition, EDTA chelated with radioactive isotopes may be precipitated in RLWs, which might need filtration.

Thus, ion-exchange resins (IERs), which are generally used in water treatment, could be a potential candidate for use in the separation and removal of EDTA in RLWs. IERs have been used as adsorbents for the purification or separation of biomaterials (e.g., proteins and amino acids) [22,23], pharmaceuticals [24], organic species (e.g., acids and alcohols) [25,26], CO_2_ [27], and metal ions [28,29]. However, EDTA removal, using IERs in an aqueous medium has been rarely reported, particularly for RLW purification. EDTA has four carboxylic acid groups, providing four negatively charged carboxylate groups in an aqueous solution. The four carboxylic acid groups can interact with positively charged functional groups on AERs, so that EDTA can be adsorbed on the AERs.

In this work, two oppositely charged IERs, such as AERs and CERs were used to compare the removal efficiency of EDTA from an aqueous solution and from a mimic of RLWs, that contained EDTA with high concentration plus Cs ions. The adsorption behavior of EDTA on the IERs was investigated under various adsorption conditions, such as adsorption time, initial EDTA concentration, pH, and the quantity of adsorbents used to elucidate the adsorption mechanism.

## 2. Experimental

### 2.1. Materials and Instrumentation

Commercial AERs (Trilite SAR12) and CERs (Trilite SCR) were supplied by Samyang Co. (Daejeon, Korea). EDTA and CsCl were purchased from Sigma-Aldrich (St. Louis, MO, USA) and used without further purification. Sodium hydroxide and hydrochloric acid were purchased from Samchun Chemical (Seoul, Korea). A high-resolution field-emission scanning electron microscope (FE-SEM, JSM-700F, Jeol, Akishima, Japan) was used to obtain the surface morphology of the IERs. Fourier transform infrared (FT-IR) spectra were obtained on a Bruker FT-IR spectrometer, using KBr pellets in the wavenumber range of 500–4000 cm^−1^. The zeta potentials of the IERs were measured using a Malvern Zetasizer Nano ZS. Total organic carbon (TOC) analysis was carried out using a Shimadzu TOC-V Series apparatus to determine the EDTA concentration before and after adsorption on the IERs. Inductively coupled plasma mass spectroscopy (ICP-MS; ELAN DRC II (PerkinElmer SCIEX, Waltham, MA, USA)) was used to determine the concentration of Cs ions before and after treatment with the IERs.

### 2.2. Determination of EDTA Concentration

Potassium hydrogen phthalate solutions (10, 100, and 1000 ppm) were used to calibrate the TOC analyzer. Aqueous EDTA solutions (0.1, 0.01, 0.001, and 0.0001 mM) were used to establish a standard curve (TOC data versus EDTA concentration). After EDTA adsorption by IERs, the aqueous EDTA solution (0.5 mL) was diluted with pure water (9.5 mL) to determine the concentration of the remaining EDTA. The EDTA concentration was calculated using a pre-established standard curve.

### 2.3. Batch Adsorption

The removal efficiency of the IERs was investigated under various conditions of adsorption time, initial EDTA concentration, and the amount of adsorbent resin (0.5–5 g/L). The EDTA concentration was adjusted from 0.01 to 0.5 mM for pure EDTA solutions, and from 0.01 to 1 mM for mixed solutions of EDTA and Cs ions with various compositions (EDTA/Cs ions (1:1, 1:2, and 1:4)). After the adsorption, an aliquot of 0.5 mL was removed from the test solution and diluted with water (9.5 mL). The amount of EDTA adsorbed on the adsorbent resin, *q* (mmol/g), and the removal efficiency (%) were calculated according to the following equations:

(1) $$q=\left(C_{0}-C\right)\times \frac{v}{m}$$

(2) $$Removal\;\left(\%\right)=\frac{\left(C_{o}-C\right)}{C_{o}}\times 100\;$$

where *C*_0_ and *C* represent the EDTA concentrations (in mM) before and after adsorption, *v* is the solution volume (L), and *m* is the weight of the IERs (g) used for EDTA removal.

## 3. Results and Discussion

### 3.1. Characterization of the IERs

**Scheme 1:**
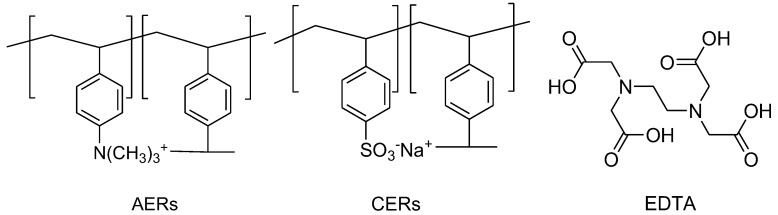
Chemical structures of ion-exchange resins (IERs) and ethylenediaminetetraacetic acid (EDTA).

**Figure 1:**
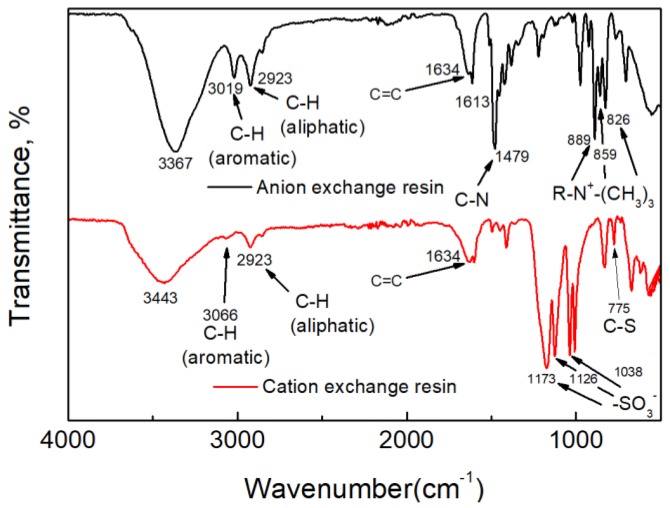
Fourier transform infrared (FT-IR) spectra of anion-exchange resins (AERs) (black) and cation-exchange resins (CERs) (red).

**Figure 2:**
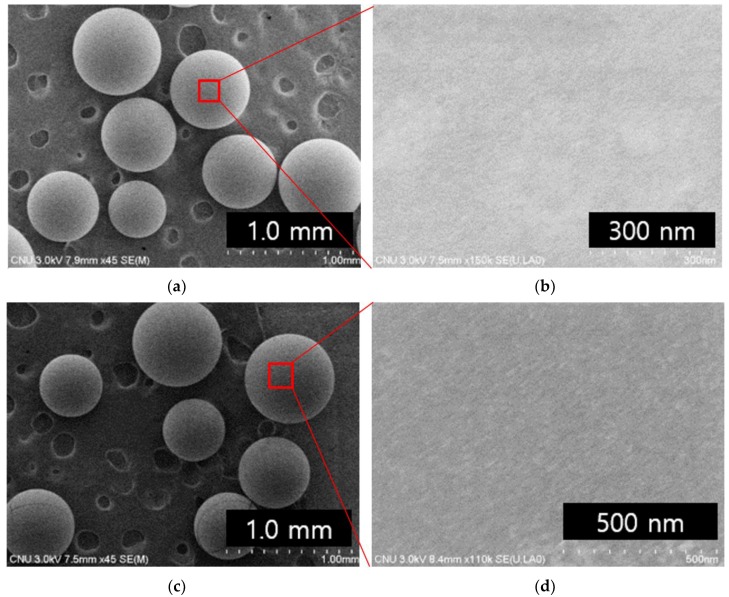
SEM images of AERs (**a**,**b**) and CERs (**c**,**d**).

IERs, including AERs and CERs, were based on a co-polymer of styrene and divinylbenzene (DVB) as a crosslinking agent with functional groups: One of tertiary amine and the other a sulfonate group, respectively (Scheme 1). The chemical structures of the IERs were confirmed by FT-IR spectroscopy (Figure 1). The characteristic bands of the aromatic C=C stretching and aliphatic C-H vibrations are shown at 1634 cm^−1^, and at 2923 cm^−1^, respectively. The characteristic band of the aromatic C–H stretching vibrations of the AERs and CERs appeared at 3019, and 3066 cm^−1^, respectively. For the AERs, the characteristic bands located at 889, 859, and 826 cm^−1^ were ascribed to R–N^+^–(CH_3_)_3_ stretching vibrations, and the characteristic band of the C–N vibration of R–N^+^–(CH_3_)_3_ appeared at 1479 cm^−1^ [30]. For CERs, the stretching vibrations of the sulfonate group are shown at 1173, 1126, and 1038 cm^−1^, and the C–S vibration appears at 775 cm^−1^. The IR assignments of IERs confirmed that the AERs and CERs had different functional groups. SEM images of both IERs showed spherical shapes with diameters in the order of hundreds of micrometers, and the surfaces were smooth, indicative of gel-type resins (Figure 2). The zeta potentials of the IERs were determined to be +75.9 mV (for AERs) and −42.5 mV (for CERs) because of their oppositely charged ionic groups, also verified from FT-IR. Because the two IERs had oppositely charged functional groups, they should show different adsorption behaviors toward the negatively charged EDTA.

### 3.2. Effect of Adsorption Time in Pure EDTA Solution

**Figure 3:**
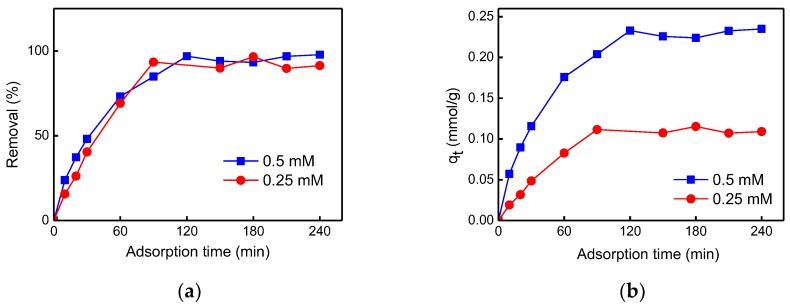
Changes in (**a**) the removal efficiency and (**b**) the adsorption capacity of AERs for ethylenediaminetetraacetic acid (EDTA) with adsorption time at EDTA concentrations of 0.25 and 0.5 mM. [AERs] = 2 g/L. pH = 7.

The changes in EDTA removal efficiency by AERs (2 g/L), with various adsorption times, were investigated: The removal was found to increase with adsorption time and became 100% saturated after about 90 min (Figure 3a). A very similar adsorption behavior was observed regardless of the EDTA concentrations (0.25 and 0.5 mM). However, the adsorption capacity (*q*_t_) was changed with EDTA concentration, showing 0.11 and 0.24 mmol/g of saturated adsorption at EDTA concentrations of 0.5 and 0.25 mM, respectively (Figure 3b). This adsorption behavior was similar to the case of using mesoporous molecular sieves in previous work [31]. It was not possible to determine the adsorption above 0.5 mM of EDTA concentration, because of the limited solubility of EDTA in water.

### 3.3. Effect of pH in Pure EDTA Solution

**Figure 4:**
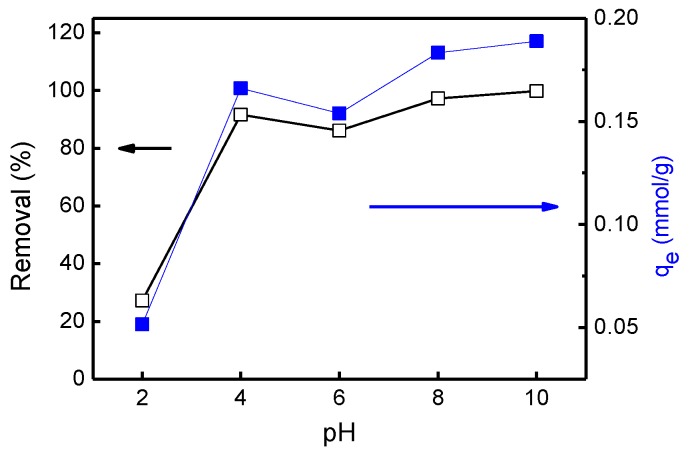
Changes in removal efficiency and adsorption capacity of AERs for EDTA at various pHs. [EDTA] = 0.5 mM; [AER] = 2 g/L; adsorption time = 5 h.

EDTA has four carboxylic acid groups and two amine groups (Scheme 1), which enable chelation of various metal ions. EDTA has four pKa values, i.e., 2.0, 2.7, 6.2, and 10.3 [32]. As a result, adsorption of EDTA by AERs can be affected by the pH of the solution. The EDTA removal efficiency by AERs was examined at various pHs (Figure 4). The adsorption efficiency of EDTA on AERs decreased significantly below pH 4, mainly because protonation of the carboxylic acid groups might hinder the chelation of metal ions. At pH values above 4, the EDTA removal efficiency approached 100%. Similar to the removal efficiency, the equilibrium adsorption capacity (*q*_e_) of AERs toward EDTA also increased with the increasing pH of the solution, suggesting that EDTA was adsorbed on AERs via a charge interaction caused by deprotonation of EDTA.

### 3.4. Effect of the Amount of Adsorbent in Pure EDTA Solution

**Figure 5:**
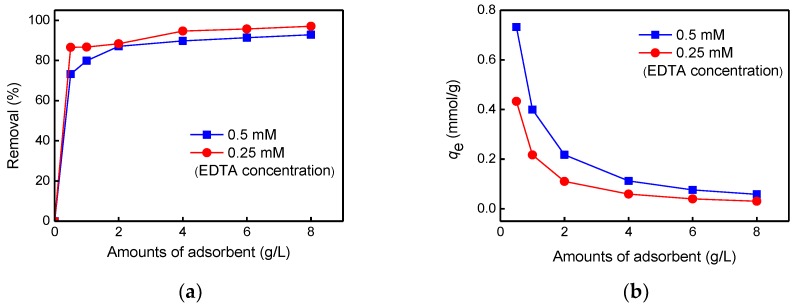
Changes in (**a**) the removal efficiency of EDTA and (**b**) the adsorption capacity of AERs at EDTA concentrations of 0.25 and 0.5 mM (pH = 7; adsorption time = 5 h).

The effects of the quantity of adsorbents on the removal and the equilibrium adsorption capacity (*q*_e_) were investigated at EDTA concentrations of 0.25 and 0.5 mM after 5 h adsorption (Figure 5). A removal efficiency of 90% was attained at these conditions when more than 4 g/L of AERs was used. The *q*_e_ decreased with an increase in the amount of AERs because the number of adsorption sites on the AER increased.

### 3.5. Effect of Adsorption Time in a Solution Mixed with EDTA and Cs Ions

**Figure 6:**
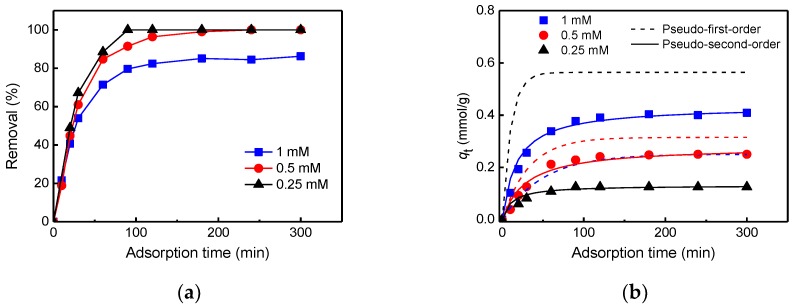
Changes in (**a**) removal efficiency of EDTA and (**b**) adsorption capacity of AERs from EDTA/Cs (1:1) with adsorption time. [AERs] = 2 g/L; [EDTA] = 0.25, 0.5, and 1 mM; pH = 7. Solid and dashed lines were fitted lines to determine adsorption order (related to Figure 12).

Large amounts of EDTA are used for the decontamination of RLWs, and thus large amounts of radioactive complexes are generated during the decontamination and decommissioning process of a nuclear facility. To simulate such radioactive complexes, a mixed solution consisting of EDTA and Cs ions, with a molar ratio of 1:1 was used (denoted as EDTA/Cs), for further adsorption experiments. The effect of the adsorption time on EDTA removal from the aqueous EDTA/Cs solution, with various EDTA concentrations, was investigated using an AERs of 2 g/L (Figure 6). EDTA alone showed limited solubility in an aqueous solution, about 0.5 mM, whereas when mixed with equivalent Cs ions, 1 mM EDTA solution could be prepared, indicating an increase in the solubility in water in the presence of Cs ions (pH 7). This solubility increase enabled us to investigate the effect of the adsorption time with EDTA concentrations of 0.25, 0.5, and 1 mM. A removal efficiency of more than 70% EDTA from the EDTA/Cs solution was achieved after 1 h of adsorption time, and the removal was saturated at 80% (for 1 mM) and at 100% (for 0.25 and 0.5 mM) after 2 h adsorption time (Figure 6a). The *q*_e_ of the AER was determined as 0.125, 0.25, and 0.40 mmol/g at EDTA concentrations of 0.25, 0.5, and 1 mM, respectively (Figure 6b). This result is similar to the case when a pure EDTA solution was used (see Figure 3). It seems that the presence of Cs ions did not affect the interaction of the AERs with EDTA, because EDTA has four carboxylic acid groups; enough to chelate Cs ions and be simultaneously adsorbed by AERs. Regarding *q*_t_, the adsorption in EDTA/Cs was enhanced to a small extent compared with the pure EDTA, presumably because of better solubility of EDTA in the presence of Cs ions.

**Figure 7:**
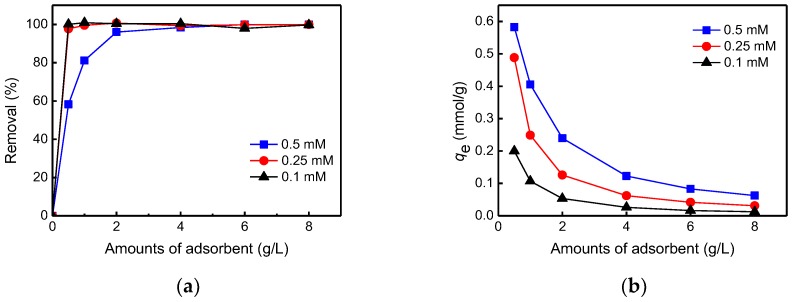
Changes in (**a**) removal efficiency of EDTA and (**b**) adsorption capacity of AERs in EDTA/Cs (1:1) with various amounts of AERs. [EDTA] = 0.1, 0.25, and 0.5 mM; pH = 7; adsorption time = 5 h.

**Figure 8:**
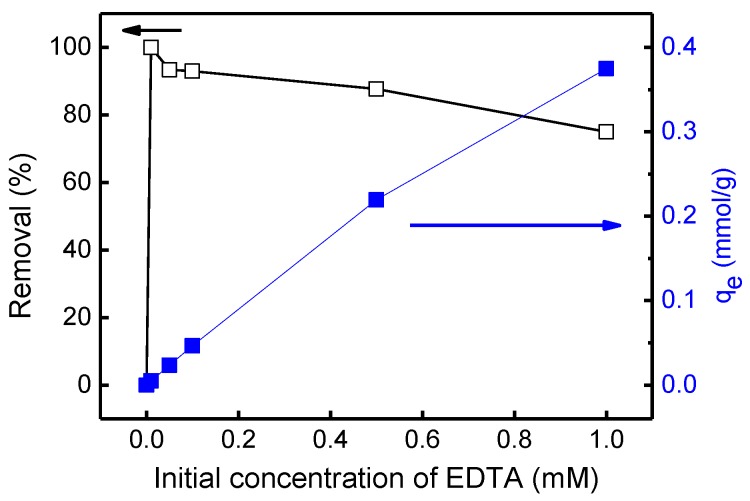
Changes in the removal efficiency of EDTA and the adsorption capacity of AERs from 1 mM EDTA/Cs (1:1) solutions containing various EDTA concentrations. [AERs] = 2 g/L; pH = 7; adsorption time = 5 h.

The removal efficiency of EDTA was found to be almost 100% when more than 2 g/L of AER was used, regardless of whether the initial EDTA concentration was 0.1, 0.25, or 0.5 mM (Figure 7). This indicates that the EDTA adsorption was more efficient in the EDTA/Cs solution than in the pure EDTA solution, in which more than 90% of the EDTA was removed from the pure EDTA solution, by using more than 4 g/L of AER after 5 h (see Figure 5). In addition, EDTA adsorption by AERs from EDTA/Cs solution in 5 h adsorption time was carried out to elucidate the effect of the initial concentrations of EDTA (Figure 8). Although the removal efficiency of EDTA using 2 g/L AER decreased with the increase in the initial EDTA concentration in EDTA/Cs solution, more than 75% of EDTA was removed from 1 mM of EDTA/Cs solution. Unlike the removal efficiency of EDTA, *q*_e_ increased continuously as the initial concentration of EDTA increased, exhibiting a maximum *q*_e_ of 0.38 mmol/g at 1 mM of the initial EDTA concentration.

**Figure 9:**
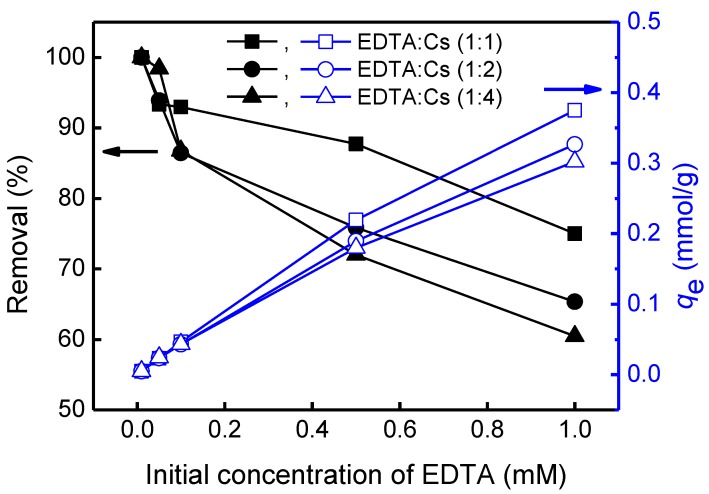
Changes in removal efficiency and adsorption capacity of EDTA using AERs in various ratios of EDTA and Cs (1:1 (■, □), 1:2 (●, ○), and 1:4 (▲, △)) with various initial EDTA concentrations (0.01–1 mM). [AERs] = 2 g/L; pH = 7; adsorption time = 5 h.

**Figure 10:**
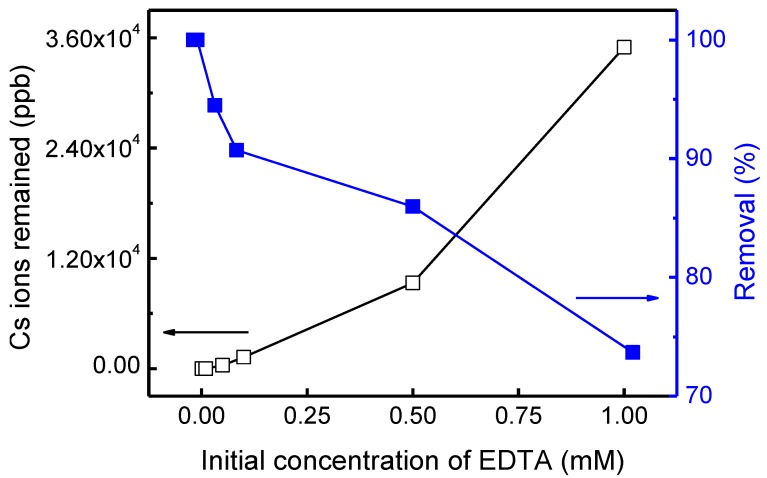
Removal efficiency of Cs ions with EDTA by AERs from EDTA/Cs (1:1) containing various initial EDTA concentrations. [AERs] = 2 g/L; pH = 7; adsorption time = 5 h.

In general, as large amounts of EDTA are frequently used for RLW decontamination, large amounts of radioactive isotopes are also present in the RLWs. Thus, it is important to investigate the effect of EDTA composition in an EDTA/Cs solution. The EDTA removal was investigated according to the Cs concentration (EDTA/Cs molar ratios of 1:1, 1:2, and 1:4) in the EDTA/Cs solutions (Figure 9). The removal efficiency of EDTA decreased with the increase in the Cs ion concentration under the same initial concentration of EDTA in solution, while, *q*_e_ increased. It was thought that the decrease in EDTA removal efficiency from EDTA/Cs solutions resulted from the changes in the charge balance of EDTA by the interaction with Cs ions. To investigate whether Cs ions were simultaneously removed from the EDTA/Cs solution during EDTA adsorption by the AERs, the remaining Cs ion concentration in the EDTA/Cs solution was determined using ICP after the adsorption. It was found that more than 70% of Cs ions were removed during the adsorption of EDTA, and 100% removal was obtained at the higher initial EDTA concentration of 1.0 mM (Figure 10). The result suggests that the Cs ions were adsorbed by the AERs as well as EDTA, indicating that these ions were already bound to EDTA. As the positively charged AERs could not adsorb isolated, positively charged Cs ions because of electrostatic repulsion. Cs ions must have been chelated with EDTA to be removed by the AERs.

**Figure 11:**
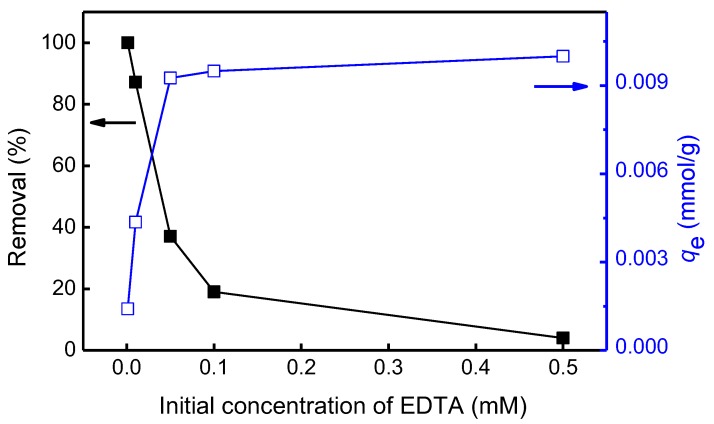
Changes in removal efficiency of EDTA (■) and adsorption capacity (□) of CERs in EDTA/Cs (1:1) containing different EDTA concentrations (0.001–0.5 mM). [CERs] = 2 g/L; pH = 7; adsorption time = 5 h.

To elucidate whether the EDTA removal was based on the charge interaction between the latter and AERs, the removal efficiency of EDTA, was investigated using CERs in an EDTA/Cs (1:1) solution with various initial EDTA concentrations. The removal efficiency of EDTA, using CERs, gradually decreased with increasing initial EDTA concentration, and eventually reached 0% (Figure 11). The *q*_e_ of CERs for EDTA removal slightly increased at a very small increment, and saturated at about 0.01 mmol/g. The adsorption performance using CERs was much lower than with AERs, demonstrating that the negatively charged CERs did not interact with the negative EDTA, unlike AERs.

### 3.6. Adsorption Kinetics

The pseudo-first- and pseudo-second-order kinetic models have been commonly used to predict adsorption kinetics. The relationship between adsorption and adsorption time was used to determine if the adsorption was a pseudo-first- or pseudo-second order kinetic model. A linear form of the pseudo-first-order model was described by Lagergren [33], as follows:

(3) $$\ln\left(q_{e}-q_{t}\right)=\ln q_{e}-k_{1}t$$

Linear data from for the pseudo-second-order model could be determined using the following equation suggested by Ho and McKay’s [34]:

(4) $$\frac{t}{q_{t}}=\frac{1}{k_{2}q_{e}^{2}}+\frac{t}{q_{e}}$$

where *q*_e_ and *q*_t_ (mg/g) are the amounts of EDTA adsorbed on the AERs at equilibrium and at various times *t* (min), and *k*_1_ (min^−1^), and *k*_2_ (g mmol^−1^ min^−1^) are the rate constants for the pseudo-first-, and pseudo-second-order equation, respectively. The term *h* (mmol g^−1^ min^−1^) means the initial adsorption rate calculated by *k*_2_*q*_e_^2^ from Equation (4) for the pseudo-second-order model.

**Figure 12:**
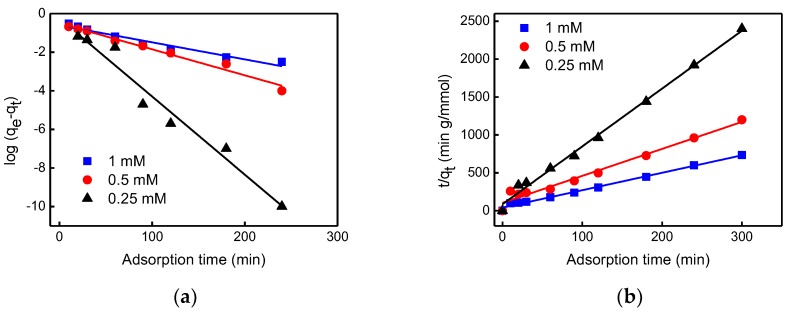
Linear fitting for (**a**) pseudo-first-order kinetic model and (**b**) pseudo-second-order kinetic model with various adsorption times. [AERs] = 2 g/L; [EDTA] = 0.25 (▲), 0.5 (●), and 1 (■) mM; pH = 7.

The linear fittings of the pseudo-first- and pseudo-second-order kinetic models are shown in Figure 12. The values of *k*_1_ and *k*_2_ for the adsorption of EDTA on AERs decreased as the initial concentration increased from 0.25 to 1.0 mM at pH 7. The adsorption of EDTA on AERs was investigated to determine the correlation coefficients (R^2^) of the two models. An R^2^ value higher than 0.97 was found from the pseudo-second-order kinetic model. From the pseudo-first-order kinetic model, the R^2^ values ranged between 0.94 and 0.97, which were lower than the values from the pseudo-second-order kinetic model. For the case of the pseudo-second-order kinetic model, the *q_e_* obtained from the experiment (*q*_e,exp_) were similar to the *q_e_* calculated from the pseudo-second-order kinetic model (*q*_e,cal_) (Table 1). Based on the higher R^2^ and the more similar *q_e_*, the pseudo-second-order kinetic model was more suitable than the pseudo-first-order kinetic model. Therefore, we believe that EDTA was adsorbed onto AERs through a chemical interaction rather than a physical one [35]. The pseudo-second-order kinetic models were applied to the experimental data described in Figure 6b, which shows the effect of adsorption time on the adsorption capacity of EDTA. The calculated values for the pseudo-second-order kinetic model (the solid lines in Figure 6b) matched well with the experimental values. The kinetics data for the adsorption of EDTA onto AER are listed in Table 1.

### 3.7. Adsorption Isotherms

Adsorption isotherms offer important information on adsorption behavior. As such, the EDTA removal was analyzed using Langmuir and Freundlich isotherms, which are representative isotherm models. The Langmuir isotherm suggests that molecules are adsorbed with a monolayer coverage of the surface of adsorbents, which means that adsorption occurs at specific sites on the surface of adsorbents [35]. The Freundlich isotherm model provides the information on adsorption behavior that occurs with the multilayer coverage of the molecules onto the surface of adsorbents [36]. The Langmuir (Equation (5)) and Freundlich isotherm equations (Equation (6)) are expressed as below:

(5) $$\frac{C_{e}}{q_{e}}=\frac{1}{bq_{max}}+\frac{C_{e}}{q_{max}}\;$$

(6) $$\log(q_{e})=\log\left(K_{F}\right)+\frac{1}{n}\log\left(C_{e}\right)$$

where *C*_e_ (mmol/L) and *q*_e_ (mmol/g) are the equilibrium concentration of EDTA and the adsorption capacity at equilibrium. *q*_max_ (mmol/g) and *b* (L/mmol) are the maximum adsorption capacity and the equilibrium adsorption constant calculated from a fitting curve. The term *n* is the parameter of the Freundlich model related to the affinity coefficient and *K*_F_ is the Freundlich constant related to the adsorption capacity regarding the equilibrium concentration (mmol/L).

**Figure 13:**
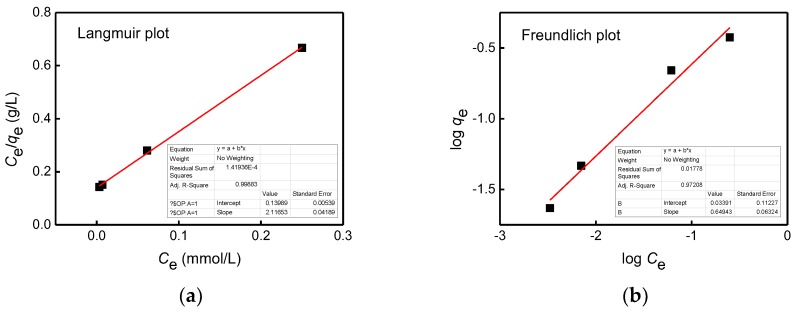
(**a**) Langmuir and (**b**) Freundlich isotherm models for the adsorption of EDTA on AERs in EDTA/Cs (1:1) mixed solution. [AERs] = 2 g/L; [EDTA] = 0.01–0.5 mM; adsorption time = 5 h.

The fitting curves for both isotherm models are shown in Figure 13. The R^2^ of the Langmuir isotherm model was found to be 0.9988, close to 1, whereas the R^2^ of the Freundlich model showed 0.9721, slightly lower than that of the Langmuir model. The Langmuir isotherm constants of *q*_max_ and *b* were determined to be 0.47 mmol/g, and 15.1 L/mmol, respectively. The Freundlich isotherm constants, 1/*n* and *K*_F_, were found to be 0.6494, and 1.081, respectively. Apparently, for the EDTA adsorption behavior on the AERs in an EDTA/Cs solution, the Langmuir isotherm model was more suitable than the Freundlich model, regarding the higher R^2^. This was good for the explanation of EDTA adsorption behavior on the AERs, which occurs by charge interaction. The isotherm constants for the adsorption of EDTA on AERs are listed in Table 2.

## 4. Conclusions

**Scheme 2:**
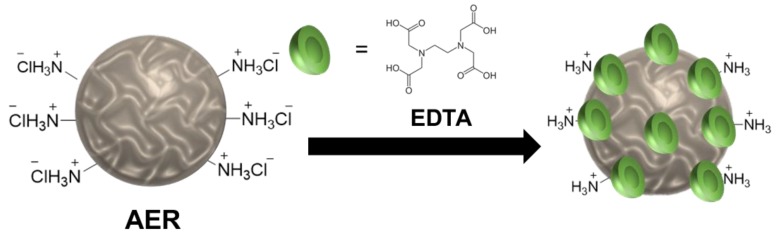
Schematic illustration of EDTA adsorption on AER.

The removal of EDTA, using positively charged AERs, was investigated in a solution containing pure EDTA and in one mixed with EDTA and Cs ions, which was a simulant of RLWs, generated during nuclear decontamination. The EDTA adsorption experiments were carried out under various conditions of adsorption time, amount of adsorbent, and initial EDTA concentration. The *q*_max_ for EDTA removal was found to be 0.47 mmol/g for initial EDTA concentrations in the range of 0.01–1 mM in the mixed EDTA/Cs solution. The EDTA adsorption on AERs occurred via chemical interaction, rather than physisorption, considering that the pseudo-second-order kinetic model was found to be suitable for the EDTA adsorption. Langmuir adsorption was found to be preferable, providing the monolayer adsorption model on the AER surface. EDTA removal, using negatively charged CERs, was also performed to verify the charge interaction between the anionic EDTA and the CERs, resulting in a considerably lower EDTA removal efficiency (Scheme 2). The results showed that the AERs were able to remove EDTA efficiently, regardless of the presence of Cs ions in the solution, indicating their potential usefulness for a separation technique for EDTA in RLWs generated from decontamination processes.

## Figures and Tables

**Table 1:** Adsorption kinetic parameters of the adsorption of ethylenediaminetetraacetic acid (EDTA) on anion-exchange resins (AERs) from EDTA/Cs solution.

*C*_0_ (mmol/L)	*q*_exp_ (mmol/g)	Pseudo-first-order			Pseudo-second-order			
		*k*_1_ (min^−1^)	*q*_e,cal_ (mmol/g)	R^2^	*k*_2_ (g mmol^−1^ min^−1^)	*q*_e,cal_ (mmol/g)	*h* (mmol g^−1^ min^−1^)	R^2^
1	0.41	0.0204	0.2503	0.9768	0.1301	0.4347	0.0074	0.9925
0.5	0.25	0.0310	0.3150	0.9487	0.1226	0.2808	0.0042	0.9721
0.25	0.13	0.0932	0.5636	0.9630	0.6827	0.1310	0.0611	0.9943

**Table 2:** Langmuir and Freundlich constants for the adsorption of EDTA on AERs.

Isotherm Models	Isotherm Constants	Value
Langmuir	*q*_max_ (mmol/g)	0.4725
	*b* (L/mmol)	15.130
	R^2^	0.9988
Freundlich	1/*n* (L/mmol)	0.6494
	*K*_F_ (mmol/g)	1.081
	R^2^	0.9721
